# Model-Based Iterative Reconstruction (MBIR) for ASPECT Scoring in Acute Stroke Patients Selection: Comparison to rCBV and Follow-Up Imaging

**DOI:** 10.3390/tomography8030104

**Published:** 2022-05-05

**Authors:** Brieg Dissaux, Mourad Cheddad El Aouni, Julien Ognard, Jean-Christophe Gentric

**Affiliations:** 1 Service d’Imagerie Médicale, CHU de la Cavale Blanche, Boulevard Tanguy Prigent, CEDEX, 29609 Brest, France; mourad.cheddadelaouni@chu-brest.fr (M.C.E.A.); julien.ognard@chu-brest.fr (J.O.); 2 Groupe d’Étude de la Thrombose Occidentale GETBO (Inserm UMR 1304), Université de Bretagne Occidentale, CHU de la Cavale Blanche, Boulevard Tanguy Prigent, CEDEX, 29609 Brest, France; 3 Laboratoire de Traitement de l’Information médicale—LaTIM (Inserm UMR 1101), Université de Bretagne Occidentale, 5 Avenue Foch, CEDEX, 29200 Brest, France

**Keywords:** stroke, CT, iterative reconstruction

## Abstract

Background: To compare a model-based iterative reconstruction (MBIR) versus a hybrid iterative reconstruction (HIR) for initial and final Alberta Stroke Program Early Ct Score (ASPECT) scoring in acute ischemic stroke (AIS). We hypothesized that MBIR designed for brain computed tomography (CT) could perform better than HIR for ASPECT scoring. Methods: Among patients who had undergone CT perfusion for AIS between April 2018 and October 2019 with a follow-up imaging within 7 days, we designed a cohort of representative ASPECTS. Two readers assessed regional-cerebral-blood-volume-ASPECT (rCBV-ASPECTS) on the initial exam and final-ASPECTS on the follow-up non-contrast-CT (NCCT) in consensus. Four readers performed independently MBIR and HIR ASPECT scoring on baseline NCCT. Results: In total, 294 hemispheres from 147 participants (average age of 69.59 ± 15.63 SD) were analyzed. Overall raters’ agreement between rCBV-map and MBIR and HIR ranged from moderate to moderate (κ = 0.54 to κ = 0.57) with HIR and moderate to substantial (κ = 0.52 to κ = 0.74) with MBIR. Overall raters’ agreement between follow-up imaging and HIR/MBIR ranged from moderate to moderate (κ = 0.55 to κ = 0.59) with HIR and moderate to almost perfect (κ = 0.48 to κ = 0.82) with MBIR. Conclusions: ASPECT scoring with MBIR more closely matched with initial and final infarct extent than classical HIR NCCT reconstruction.

## 1. Introduction

Brain parenchymal damages can be assessed by computed tomography (CT) and magnetic resonance imaging (MRI). They allow to guide therapeutic strategy; even if MRI can be used in this indication the rapidity, the availability, resolution, possibility of an adequate vessels analysis (CTA), and possibility to perform a perfusion (CTP) led to the wide use of CT in this indication [1,2]. Non-contrast CT (NCCT) always the first step of this protocol and remains of great importance because of the early stroke signs leading to the assessment of the viability of the brain. Early ischemic changes are difficult to assess [3]. On the contrary, late ischemic changes are easier to assess especially on NCCT follow-up imaging. The Alberta Stroke Program Early CT Score (ASPECTS) was designed to summarize these changes in one global score (0–10). The ASPECT score is used to quantify the extent of an ischemic stroke occurring in the middle cerebral artery. This territory is divided into 10 zones and when early signs of ischemia are visible in a zone, 1 point is removed. Even if one major drawback of the ASPECT score is the non-perfect inter-observer agreement, this approach is used widely in practice and in trials [4,5,6].

New iterative methods have been developed to improve image quality and to permit radiation dose reduction [7,8,9]. Two basic categories can be distinguished: hybrid iterative reconstructions (HIRs) and Model-based iterative reconstructions (MBIRs). Adaptative Iterative Dose Reduction 3D (AIDR3D) is an HIR designed to reduce radiation doses while preserving image quality and performs iteration in both the image and the raw data domain [10]. MBIRs perform iterative reconstructions consisting of forward and backward reconstructions steps only in the raw data domain [10]. Whereas HIRs are widely used, progress in computer software and hardware technology reduced the time required for MBIRs and made them recently clinically usable. Forward projected model-based Iterative Reconstruction SoluTion Low-Contrast-Dynamic (FIRST-LCD) (Canon Medical System, Tokyo, Japan) is an MBIR solution designed to provide better low-contrast detectability and may, therefore, help in detecting early ischemic changes [11,12,13,14]. A few studies have reported in vitro or with a small number of patients the benefit of iterative reconstructions in acute ischemic stroke (AIS) [11,12,13,14].

The hypothesis of the present work was that scoring initial ASPECT with FIRST-LCD might better predict the final ASPECT score than with AIDR3D. The second hypothesis was that scoring initial ASPECT with FIRST-LCD might better match with rCBV ASPECTS than AIDR3D. The purpose of this study was to assess the detectability of early ischemic changes on baseline NCCT with FIRST-LCD (FIRST-LCD ASPECTS) compared to AIDR3D (AIDR3D ASPECTS) using follow-up imaging (final ASPECTS) and rCBV ASPECTS as surrogates for final and initial infarct extent respectively. To answer these questions, the diagnostic performance of these reconstruction techniques will first be compared to the final ASPECTS and rCBV ASPECTS. Then, measures of agreement between these reconstruction techniques and the final ASPECTS final and rCBV ASPECTS will be computed.

## 2. Materials and Methods

### 2.1. Subsection Participant Selection

This single-center study was conducted between April 2018 and October 2019. The retrospective protocol was approved by the Ethics Committee of CERIM (CRM-1910-033). According to national laws, written informed consent was waived. We searched the electronic medical records of our institution to identify patients who underwent CT protocol for AIS with follow-up imaging performed within 7 days to assess final ASPECTS. All the participants had their initial CT in our institution and this CT was used for the assessment of urgent revascularization using thrombolysis and/or thrombectomy.

### 2.2. Image Acquisition and Reconstruction

All patient underwent the same CTA-CTP protocol (CT Aquilion One Genesis—Canon Medical Systems). Our detailed protocol has been previously published [15]. Briefly, acquisitions were performed on a 320-row area detector CT Aquilion ONE Genesis (Canon Medical Systems). Main acquisitions were performed using the following parameters: 320 × 0.5 mm collimation, 220 mm field of view, 80 kV tube voltage, and 0.75 s rotation time. A NCCT scan was performed first (300 mA), leading to two different reconstructions (AIDR3D and FIRST-LCD). Then, CTP was performed after a bolus test (injection of 60 mL contrast medium at 5 mL/s followed by 50 mL of saline flush solution at 5 mL/s. An rCBV map from CTP was processed with VitreaWorkStation (Canon Medical Systems) and could show infarct core (rCBV < 41%). Follow-up imaging was performed with NCCT or MRI. NCCT was performed on a Somatom Def AS 64 and an Aquilion One Genesis CT with routinely used reconstruction (HIR: SAPHIRE or AIDR3D respectively). MRI consisted among others of a diffusion weighted imaging (b1000) performed on an Optima MR450W or an Achieva 3T scan.

### 2.3. Readings of FIRST-LCD and AIDR3D ASPECTS on Baseline NCCT

First, four radiologists with various experience in Neuroradiology (JCG: 10 years, JO: 7 years, BD: 5 years and MCEA: 3 years) reviewed, independently, anonymized baseline NCCT, in a randomized fashion, for both reconstructions (AIDR3D and FIRST-LCD separately). Readers were blinded to patient’s clinical outcome and any other imaging and assessed FIRST-LCD ASPECTS and AIDR3D ASPECTS for both hemispheres.

Next, two readers (JO and BD) rated in consensus, in the same manner, FIRST-LCD and AIDR3D ASPECTS for both hemispheres.

### 2.4. Consensus Readings of Final and rCBV ASPECTS

For a third analysis (4 weeks after the second reading session to avoid recall bias), two readers (JO and BD) assigned in consensus an ASPECT score on the baseline CTP rCBV map (rCBV ASPECTS) and the final follow-up imaging (MR or CT: final ASPECTS).

At that time, the readers were aware of the clinical data and outcome. The rCBV map permitted to establish rCBV ASPECTS and follow-up imaging permitted to establish the final ASPECTS, which served as surrogates for initial and final infarct extent, respectively. Abnormal rCBV was defined as unequivocal reduction compared to the corresponding region in the unaffected hemisphere [16].

### 2.5. Data Analysis

Standard analysis of the cohort was delivered through a descriptive analysis of the variables.

Data analysis aimed at comparing the reading of ASPECTS for each hemisphere (given readers were blinded to all clinical data) using the two reconstruction techniques FIRST-LCD and AIDR3D to assess the initial and final infarct extent. This approach was used in order not to ignore possible errors related to the reading with these reconstruction techniques which could have led to a misidentification of the pathological side.

The diagnostic performances of these two reconstruction techniques were evaluated by opposing the reliability of these techniques face to face. First, we compared separately final ASPECTS and initial rCBV ASPECTS with initial FIRST-LCD and AIDR3D ASPECTS in the consensus analysis. Every healthy hemisphere was rated an ASPECTS of 10. Therefore, statistical analyses were performed for FIRST-LCD ASPECTS < 10 and then AIDR3D ASPECTS < 10 on initial NCCT and rCBV ASPECTS < 10. Mean differences between these scores were calculated. For visual analysis, difference plots were provided. Inter-technique agreements between reconstruction techniques and references for all ASPECTS, ASPECTS < 10, and in a subitems analysis were performed (consensus analysis).

Second, this reliability was represented by the agreement between the evaluation of the FIRST and AIDR3D techniques by each reader and final and rCBV ASPECTS separately. Statistical analyses were performed for final ASPECTS < 10. The reproducibility of scoring ASPECT (analysis for final ASPECTS < 10) using either FIRST-LCD or AIDR3D was assessed by the inter-reader agreement between the four raters for the total ASPECTS and for each of its ten subitems (kappa), separately for each technique.

The agreement measures consisted in: kappa statistics (adapted: Cohen’s or Fleiss’, weighted or not), Intraclass Correlation (ICC). For a visual analysis of the agreements, correlation plots were provided. The weighted kappa was calculated by using a predefined table of weights (quadratic, the higher the disagreement, the higher the weight). A bootstrap procedure was performed to estimate 95% CI of kappa and to perform statistical tests. Interpretation followed the proposed standards of Landis and Koch: 0–0.20 (slight); 0.21–0.40 (fair); 0.41–0.60 (moderate); 0.61–0.80 (substantial); and 0.81–1.00 (almost perfect). A value of *p* < 0.05 was considered as significant (95% CI). Statistical analysis was performed using R 3.6.3 (2020 The R Foundation for Statistical Computing).

## 3. Results

### 3.1. Participants

The images of 147 participants were analyzed leading to the study of 294 hemispheres. The cohort included 79 (54%) male and 68 (46%) female, with an average age of 69.59 ± 15.63 (Standard Deviation (SD)) years. The median baseline National Institutes of Health Stroke Scale (NIHSS) score was 8 (Interquartile Range (IQR) 3–18) (data for N = 134/147). Twenty-seven (18%) patients benefited from intravenous thrombolysis (IVT), 37 (25%) from mechanical thrombectomy (MT), and 20 (14%) from both MT and IVT. Seventy-eight percent (N = 114) of the cohort was followed-up by CT-scanner, and the rest (22%, N = 32) by MRI, at a median time of 1 day (IQR 1–3). The median NIHSS score at discharge was 2 (IQR 0–7) with a median negative shift of 3 (0–8), and 3 patients died during hospitalization (data for N = 132/147).

### 3.2. ASPECTS Distribution

The distribution of ASPECTS within the cohort was described according to follow-up imaging (final ASPECTS), as a final infarct extent surrogate. The distribution skewed toward higher ASPECTS (smaller infarcts), with 14 participants demonstrating ASPECTS inferior or equal to 5. The median final ASPECTS was 8 (IQR 6–9) for the 91/294 (31%) ASPECTS < 10.

### 3.3. Comparison of Final and rCBV ASPECTS with FIRST-LCD and AIDR3D ASPECTS

**Figure 1:**
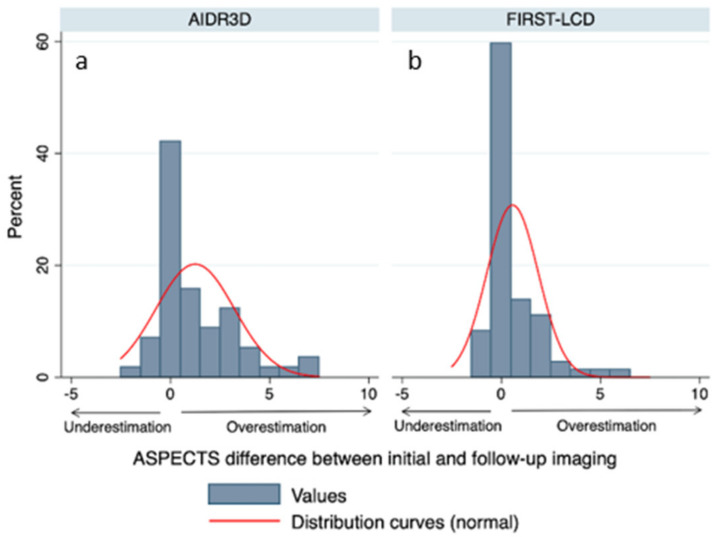
ASPECTS difference between initial FIRST-LCD (**b**) and AIDR3D (**a**) and follow-up imaging (final ASPECTS). The narrower the distribution curve, the higher the distribution curve near 0. FIRST-LCD ASPECTS (**b**) more closely match the final ASPECTS than AIDR3D ASPECTS (**a**).

There was a mean difference between FIRST-LCD and final ASPECTS of 0.09 [−0.28, 0.47 95% CI] and a mean difference between AIDR3D and final ASPECTS of 1.1 [0.57, 1.62 95% CI]. These differences in ASPECTS were significantly different (difference FIRSTLCD-AIDR3D: −1.01 [−1.63, −0.38 95% CI] *p* = 0.002). Figure 1 provides differences between initial ASPECTS and final ASPECTS.

**Figure 2:**
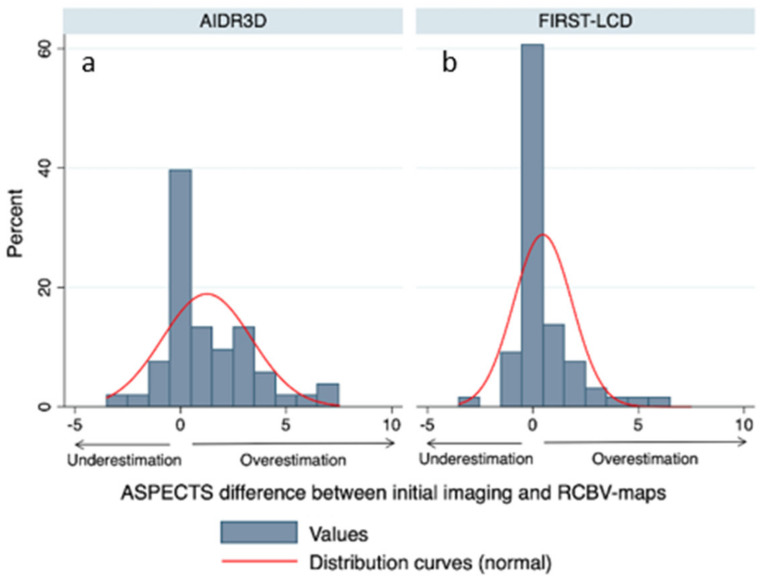
ASPECTS difference between initial FIRST-LCD (**b**) and AIDR3D (**a**) and rCBV-map (rCBV ASPECTS). The narrower the distribution curve, the higher the distribution curve near 0, and the smaller the difference in ASPECT scoring between a reconstruction technique (FIRST-LCD or AIDR3D) and rCBV ASPECTS. FIRST-LCD ASPECTS (**b**) more closely match the rCBV ASPECTS than AIDR3D ASPECTS (**a**).

There was a mean difference between FIRST-LCD and rCBV ASPECTS of 0.58 [0.14, 1.02 95% CI] and a mean difference between AIDR3D and rCBV ASPECTS of 1 [0.55, 1.44 95% CI]. These differences in ASPECTS were not significantly different (difference FIRST-LCD-AIDR3D: −0.41 [−1.04, 0.21 95% CI] *p* = 0.19). Figure 2 provides differences between initial ASPECTS and rCBV ASPECTS.

### 3.4. Agreements between FIRST-LCD and AIDR3D ASPECTS according to Final and rCBV ASPECTS: Readers Analysis

**Figure 3:**
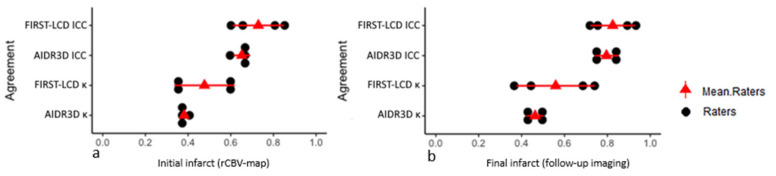
Dotplot showing comparative agreements between AIDR3D and FIRST-LCD according to rCBV-map (**a**) and follow-up imaging (**b**). Means raters agreements were computed for visual purposes.

Comparative agreements (kappa, ICC) between FIRST-LCD and AIDR3D according to rCBV map and follow-up imaging are shown in a dotplot (Figure 3).

For the assessment of final infarct extent, overall raters’ agreement (kappa) between final ASPECTS and FIRST-LCD and then AIDR3D ranged from fair to substantial (κ = 0.37 to κ = 0.74) with FIRST-LCD and from moderate to moderate (κ = 0.42 to κ = 0.50) with AIDR3D (Figure 3).

For the assessment of initial infarct extent, overall raters’ agreement (kappa between rCBV map and FIRST-LCD and then AIDR3D ranged from fair to substantial (κ = 0.34 to κ = 0.61) with FIRST-LCD and from fair to moderate (κ = 0.36 to κ = 0.41) with AIDR3D (Figure 3). The reader’s agreements were more dispersed with FIRST-LCD than with AIDR3D. Table 1 provides inter-technique agreement with AIDR3D and FIRST-LCD for the ASPECT score and in a subitem analysis. Ischemic changes are more difficult to detect in Internal capsule and M4 with FIRST-LCD than AIDR3D.

### 3.5. Inter-Reader Agreement

Table 2 provides inter-reader agreements with FIRST-LCD and AIDR3D for ASPECT scoring and in a subitem analysis. Inter-reader agreements were similar between FIRST-LCD and AIDR3D.

**Figure 4:**
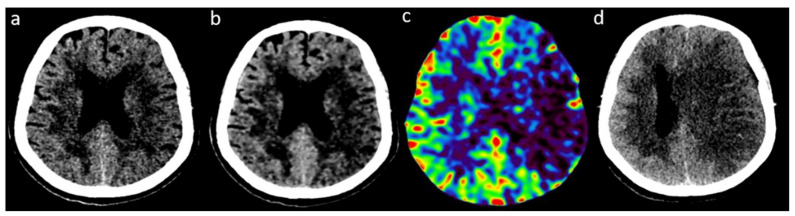
Illustrative case of an acute ischemic stroke with clot in left M1. (**a**): NCCT with AIDR3D; (**b**): NCCT with FIRST-LCD with the same windowing); (**c**): rCBV-map; (**d**): NCCT follow-up imaging (24 h). (**a**,**b**) Hypodensity in left middle cerebral artery territory. (**c**) Hypoperfusion in the same territory and (**d**) Ischemic infarction with mass effect.

Figure 4 demonstrates an illustrative case of an acute ischemic stroke with clot in left M1.

**Figure 5:**
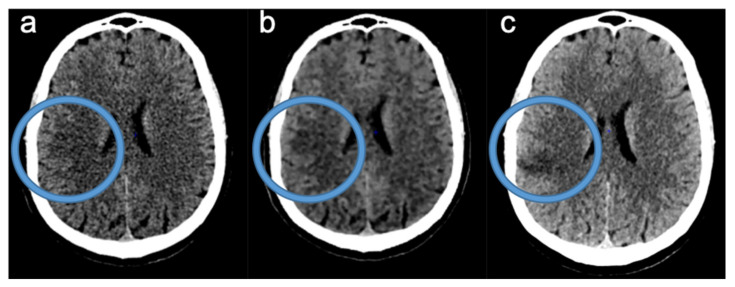
Illustrative case with a difference between ASPECTS with FIRST-LCD and AIDR3D. (**a**): NCCT with AIDR3D; (**b**): NCCT with FIRST-LCD with the same windowing); (**c**): NCCT follow-up imaging. Example of an acute ischemic stroke in the right cerebral arterial territory detected on (**b**) with ASPECT 9/10, whereas (**a**) was scored ASPECT 10/10. (**c**) Confirms the acute ischemic stroke.

Figure 5 shows an illustrative case with a difference between ASPECTS with FIRST-LCD and AIDR3D.

## 4. Discussion

This study compared, in AIS, the ASPECT scoring of initial NCCT with two reconstruction techniques (FIRST-LCD and AIDR3D) to initial brain perfusion data (rCBV map) and follow-up imaging. Initial brain perfusion data and follow-up imaging were used as surrogate for initial infarct extent and final infarct extent, respectively. For the assessment of the final infarct extent, initial ASPECTS with FIRST-LCD more closely matched with final ASPECTS (significant, difference FIRST-LCD-AIDR3D: −1.01 [−1.63, −0.38 95% CI] *p* = 0.002) than AIDR3D ASPECTS. Overall raters’ agreement (kappa) for all ASPECTS between follow-up imaging and reconstructions techniques ranged from moderate to moderate (κ = 0.55 to κ = 0.59) with AIDR3D and moderate to almost perfect (κ = 0.48 to κ = 0.82) with FIRST-LCD.

For the assessment of initial infarct extent, initial ASPECTS with FIRST-LCD more closely matched with rCBV ASPECTS (not significant, difference FIRST-LCD-AIDR3D: −0.41 [−1.04, 0.21 95% CI] *p* = 0.19) than AIDR3D ASPECTS. Overall raters’ agreement (kappa) for all ASPECTS between rCBV map and reconstructions techniques ranged from moderate to moderate (κ = 0.54 to κ = 0.57) with AIDR3D and moderate to substantial (κ = 0.52 to κ = 0.74) with FIRST-LCD.

Reading of ASPECTS with FIRST-LCD reached higher agreements with rCBV and follow-up imaging ASPECTS than with AIDR3D. However, the distribution of agreements was more spread out with FIRST-LCD than with AIDR3D.

As already reported by Iyama et al., we assume that the readers’ experience with these reconstructions may have smoothed the performance [17].

Current guidelines integrate ASPECTS in the decision-making process for intervention in patients with AIS. FIRST-LCD was supposed to provide better low contrast detectability, and therefore, might help in detect subtle ischemic changes. As already pointed out by other works, a major drawback of the ASPECTS evaluation is its moderate inter-reader agreement, especially in a subitem analysis [18,19,20]. Therefore, we aimed to assess the impact of FIRST-LCD on the inter-reader agreement. Despite some limitations, we used rCBV map as a surrogate for initial infarct assessment, because applying ASPECTS to Regional Cerebral blood volume map (rCBV map) derived from CTP has been reported to reduce variability and improve the detection of early ischemic changes in comparison to NCCT ASPECTS [19]. In our study, inter-reader agreement did not seem to be improved with FIRST-LCD but appeared more dispersed, possibly due to different experiences with these reconstructions [17]. In our work, ICC were always higher than kappas. Indeed, ICC have been reported to artificially make ASPECTS look reliable, whereas kappas that correct for chance do not [6]. Few studies reported a potential interest of FIRST-LCD in reducing radiation dose while preserving the contrast-to-noise ratio, whereas others did not [21,22,23,24]. The potential of iterative reconstruction for the diagnosis of small fossa stroke has also been reported [25]. In the present work, we assessed in an objective manner the potential added-value of such reconstruction (MBIR) versus routinely used reconstruction (AIDR3D) in the ASPECT scoring in a non-selected population of stroke patients. Because these patients may benefit from a treatment or not, we assessed, in addition to follow-up imaging, the rCBV map from baseline CTP [16]. The readers were blinded to clinical data and especially of the affected side. This may seem artificial but was deemed useful to assess the technique, especially to detect early and/or small ischemic changes.

Limitations of our study include, first, the study’s retrospective and monocentric design. Second, the distribution skewed toward higher ASPECTS (smaller infarcts), with 14 patients demonstrating ASPECTS inferior or equal to 5. However, analyses were performed in ASPECTS < 10. In addition, ASPECTS distribution of our population study appears similar to previous works [18,19]. Moreover, this may have limited a possible bias of over-scoring with FIRST-LCD. Third, the delay between follow-up imaging and baseline NCCT might be a limitation. However, we recorded a median delay of 1 day (IQR 1–3) and we assessed initial rCBV ASPECTS. Fourth, the difference in ASPECTS between initial NCCT and follow-up imaging could also be explained by patient cares with early reperfusion or not. This is why we also assessed initial rCBV ASPECTS. Furthermore, initial FIRST-LCD ASPECTS more closely matched with final ASPECTS than with rCBV ASPECTS. Fifth, because of the exploratory design of the present study, we acknowledge the follow-up imaging provided with CT and MRI. However, each patient was his own control and initial rCBV ASPECTS was provided with rCBV map for all patients. Moreover, despite different CTs were used for follow-up imaging, with different HIR, this does not seem too impactful to us because contrary to early ischemic changes, late ischemic changes are much easier to assess. Sixth, a potential drawback might be the comparison of FIRST-LCD versus AIDR3D that is an HIR and not FBP. However, we believe this provides a better understanding of the potential benefit of this kind of innovation compared to what is used today. Finally, our work only assessed one MBIR (FIRST-LCD) that aims to provide better low contrast detectability and did not evaluate the potential dose reduction of FIRST-LCD in-vivo because that would require two different acquisitions for the same patient. Further studies, ideally prospective and multi-centric, will be able to complete these data with, if possible, a single modality to assess the final ASPECT (MRI or CT). In addition, further studies may determine whether ASPECTS readings using these reconstructions could lead to changes in treatment indications.

## 5. Conclusions

These data support the improvement of CT imaging in acute stroke by new reconstructive techniques. Overall, for patients undergoing CT for acute ischemic stroke, the determination of the ASPECTS with FIRST-LCD more closely matched with initial and final infarct extents than classical NCCT reconstruction.

## Figures and Tables

**Table 1:** Inter-technique agreement.

		AIDR3D				MBIR				
			Kappa	95%CI		Correlation	Kappa	95%CI		Correlation
NCCT or DWI Control	ASPECTS *	All	0.59	0.52	0.65	0.74	0.81	0.78	0.83	0.92
		ASPECTS < 10	0.42	0.33	0.52	0.67	0.63	0.54	0.73	0.75
	SUBITEMS **	Caudate	0.49	0.28	0.71	0.55	0.65	0.46	0.83	0.66
		Insula	0.72	0.61	0.84	0.73	0.85	0.77	0.94	0.86
		Internal Capsule	0.36	0.07	0.66	0.47	0.34	0.05	0.63	0.40
		Lenticular	0.60	0.43	0.76	0.63	0.79	0.67	0.91	0.79
		M1	0.68	0.51	0.85	0.68	0.76	0.62	0.91	0.77
		M2	0.64	0.48	0.79	0.65	0.82	0.70	0.93	0.82
		M3	0.43	0.15	0.72	0.53	0.68	0.46	0.90	0.70
		M4	0.58	0.37	0.79	0.60	0.71	0.53	0.89	0.72
		M5	0.51	0.37	0.64	0.54	0.85	0.76	0.93	0.85
		M6	0.31	0.06	0.56	0.33	0.69	0.51	0.87	0.69
rCBV Acute CTP	ASPECTS *	All	0.55	0.53	0.57	0.68	0.72	0.67	0.79	0.85
		ASPECTS < 10	0.34	0.30	0.42	0.51	0.51	0.48	0.63	0.57
	SUBITEMS **	Caudate	0.50	0.25	0.76	0.52	0.55	0.32	0.78	0.55
		Insula	0.52	0.37	0.68	0.53	0.62	0.48	0.76	0.63
		Internal Capsule	0.49	0.07	0.92	0.51	0.66	0.30	1,00	0.67
		Lenticular	0.55	0.35	0.75	0.55	0.65	0.49	0.82	0.66
		M1	0.62	0.45	0.80	0.63	0.76	0.62	0.89	0.76
		M2	0.46	0.27	0.65	0.46	0.65	0.49	0.81	0.65
		M3	0.36	0.06	0.66	0.42	0.66	0.42	0.89	0.66
		M4	0.49	0.27	0.71	0.52	0.44	0.22	0.65	0.45
		M5	0.50	0.36	0.64	0.52	0.81	0.72	0.90	0.81
		M6	0.31	0.03	0.60	0.31	0.55	0.33	0.78	0.58

Inter-technique agreement between reconstruction techniques and references, provided by Cohen’s Kappa (two ratings: studied technique AIDR3D or MBIR, and reference ones: CT Perfusion or control imaging) quadratically weighted * for ASPECTS and unweighted ** for subitems assorted of its 95% Confidence Interval (95%CI), and the correlation coefficient (Spearman Rhô, pairwise). All readings are consensus.

**Table 2:** Inter-reader agreement.

Agreement Parameter	Items	AIDR3D	FIRST-LCD
Kappa *	All	0.31	0.26
ICC	All	0.93	0.92
Kappa *	Caudate	0.38	0.25
	Insula	0.63	0.69
	Internal Capsule	0.44	0.31
	Lenticular	0.53	0.38
	M1	0.62	0.57
	M2	0.53	0.58
	M3	0.40	0.48
	M4	0.60	0.65
	M5	0.53	0.40
	M6	0.47	0.58

Inter-reader agreement with AIDR3D and FIRST-LCD for ASPECT score and in a subitem analysis. * Fleiss Kappa, four raters, unweighted.

## Data Availability

Restrictions apply to the availability of these data. Data are available on reasonable request from the authors with the permission of the local Ethic Committee.
